# Fundamentals and applications of metal nanoparticle- enhanced singlet oxygen generation for improved cancer photodynamic therapy

**DOI:** 10.3389/fchem.2022.964674

**Published:** 2022-07-22

**Authors:** Blassan P. George, Alexander Chota, Paromita Sarbadhikary, Heidi Abrahamse

**Affiliations:** Laser Research Centre, Faculty of Health Sciences, University of Johannesburg, Johannesburg, South Africa

**Keywords:** cancer, metallic nanoparticles, nanotechnology, photodynamic therapy, photosensitizers, photothermal therapy, singlet oxygen, surface plasmon resonance

## Abstract

The introduction of nanotechnology in the field of Photodynamic Therapy (PDT) has proven to have great potential to overcome some of the challenges associated with traditional organic photosensitizers (PS) with respect to their solubility, drug delivery, distribution and site-specific targeting. Other focused areas in PDT involve high singlet oxygen production capability and excitability of PS by deep tissue penetrating light wavelengths. Owing to their very promising optical and surface plasmon resonance properties, combination of traditional PSs with plasmonic metallic nanoparticles like gold and silver nanoparticles results in remarkably high singlet oxygen production and extended excitation property from visible and near-infrared lights. This review summarizes the importance, fundamentals and applications of on plasmonic metallic nanoparticles in PDT. Lastly, we highlight the future prospects of these plasmonic nanoengineering strategies with or without PS combination, to have a significant impact in improving the therapeutic efficacy of cancer PDT.

## 1 Introduction

Cancer is a medical condition characterized by unregulated proliferation of abnormal cells and their metastatic abilities which allow them to spread from the site of origin to distant body tissues and organs, which if left untreated, leads to several serious medical complications and eventually death (Fares et al., 2020; Majidpoor and Mortezaee, 2021; Hanahan, 2022; Pavlova et al., 2022). According to the International Agency for Research on Cancer’s GLOBOCAN projections for 2020, global cancer incidence rate was reported to be 19.3 million cases in 2020, with projections of 30.2 million cases by 2040. The mortality rate is also anticipated to rise from 9.96 million of 2020 to 16.3 million by 2040 (Sung et al., 2021). The rise in the incidence and death rate can be ascribed to a number of factors such as age, alcohol consumption, familial history, tobacco smoking, viruses, chemicals, and consistent exposure to radiations (e.g., ultraviolet radiation) (Danaei et al., 2005; Vineis and Wild, 2014; Whiteman and Wilson, 2016; Lewandowska et al., 2019).

**FIGURE 1:**
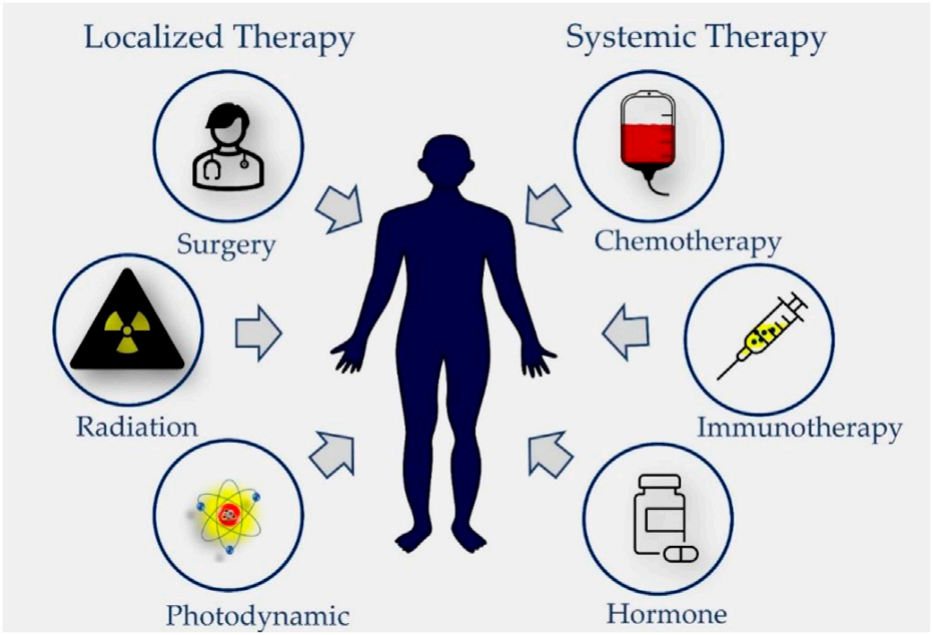
Different treatment modalities used in cancer therapy. Locoregional treatment modalities like surgery and radiotherapy are used to treat early-stage solid tumors and Photodynamic Therapy is applicable for treatment of superficial cancers. Systemic therapies such as targeted and non-targeted cytotoxic chemotherapy, hormone therapy, and immunotherapy are either used as monotherapy or in combinations or adjuvant treatment strategy for early or locally advanced solid tumors as well as palliative therapy for metastatic solid tumors.

The treatment of cancer is commonly categorized as being either curative or palliative dependent on the location and stage of the cancer (Neugut and Prigerson, 2017; Miller et al., 2019). In general, based on their target and mechanisms of action the anticancer treatment modalities are classified into two major groups: localized, and systemic (Figure 1) and each of them differs on basis of their benefits as well as their limitations which varies from mild to severely adverse side effects (Bidram et al., 2019; Debela et al., 2021; Pestana and Ibrahim, 2021).

**FIGURE 2:**
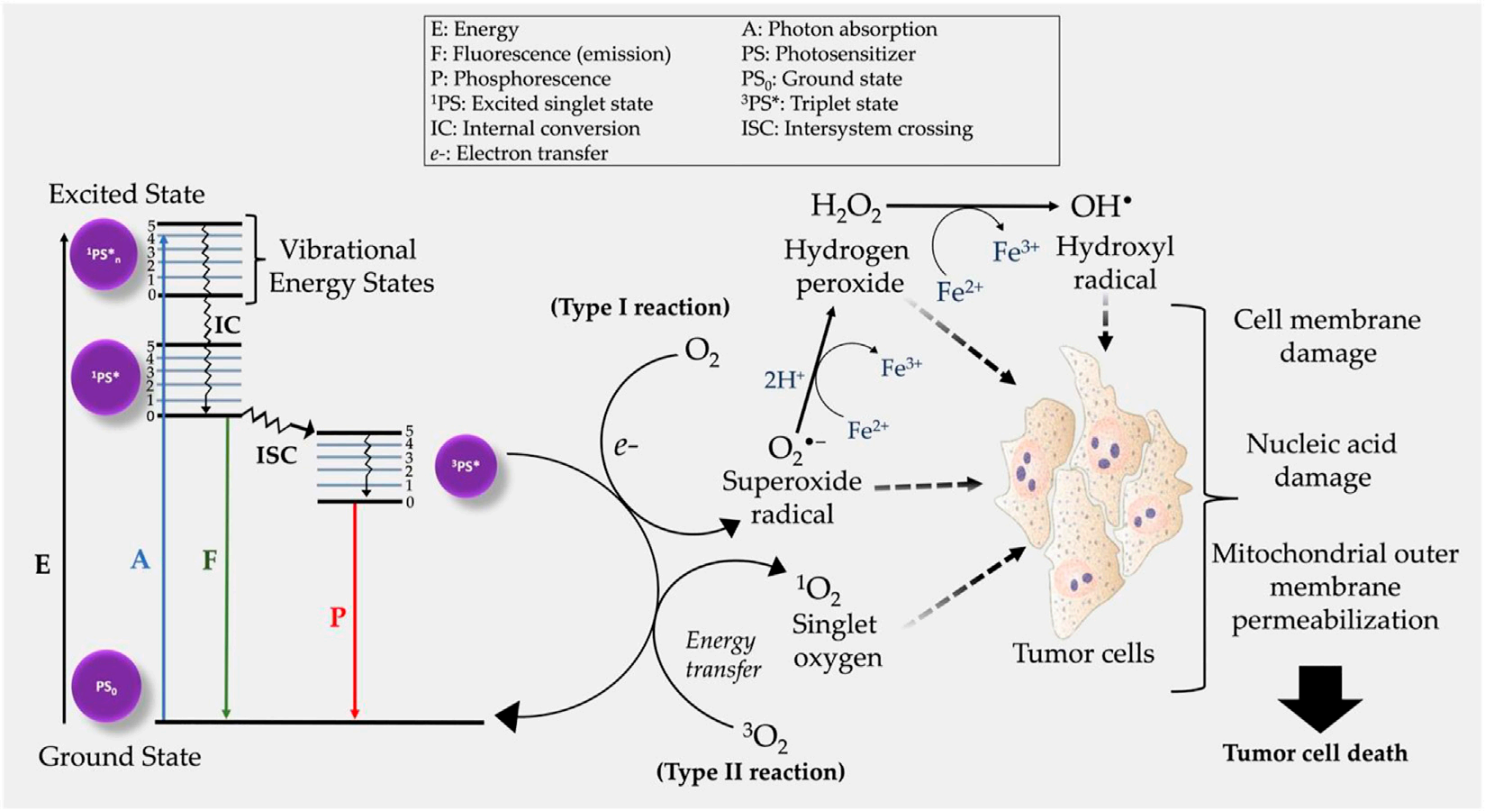
Modified Jablonski diagram demonstrating the mechanism of action of photodynamic therapy. Following the generation of intracellular ROS, tumor cell death is initiated post oxidative stress on several cell organelles.

Photodynamic therapy (PDT) is a relatively novel and clinically approved localized treatment modality which make use of electromagnetic radiation in the Visible and Near Infrared energy region to excite photosensitizing drugs known as photosensitizers (PSs) (Chen et al., 2022). As represented in Figure 2, in an excited state PS, the interactions of photosensitizers with molecular oxygen (O_2_) and surrounding biomolecules results in the generation of highly toxic Reactive Oxygen Species (ROS) (Zhou et al., 2016; Dos Santos et al., 2019; Yanovsky et al., 2019; Li et al., 2020; Gunaydin et al., 2021). On molecular and cellular level, the generated ROS interacts with cellular organelles such as the mitochondria, endoplasmic reticulum, peroxisomes, and the nucleus and causing their structural and functional impairments to result into tumor cell death (Figure 2). However, as conventional PS do not accumulate in cell nuclei, thus PDT avoids the development of genetically resistant cells due to its very low potential of causing DNA damage, mutations, and carcinogenesis (Agostinis et al., 2011). As compared to conventional therapies PDT offers several advantages as anticancer treatment which include less invasiveness, minimum induced side effects, tumor selectivity due to the preferential accumulation of PSs and targeted light irradiation of tumor lesions, repetition of treatment several times without inducing resistance in cancer cells, no scar formation after healing, cost effectiveness. However, like any other therapy, PDT also suffers from the limitations such as PS targetability, delivery and accumulation in tumor mass, skin photosensitivity, not applicable for metastatic cancers, ineffective for large or deep-seated tumors due to the inadequate penetration of light in tissues, and decreased PDT efficacy under tumor hypoxia conditions, all of which hampers the overall therapeutic outcome (Correia et al., 2021; Gunaydin et al., 2021).

Mechanistically PDT mediated ROS generation is facilitated by triplet excited PS state (^3^PS^*^), resulting into two main types of reactions i.e., type I and type II photochemical reactions (Figure 2). Type I PDT reactions, involve either the donation or acquisition an electron thereby forming radical anions or radical cations (Abrahamse and Hamblin, 2016). The generated ROS radicals (e.g., O_2_^•−^) may undergo further reduction and oxidation (redox) reactions, and produce other intermediate ROS e.g., hydrogen peroxide (H_2_O_2_), and hydroxyl radical (OH^•^) (Collin, 2019; Chen et al., 2022). Alternatively, PDT effects can initiate the activation/or induction of type II photochemical reactions in which singlet oxygen (^1^O_2_) is generated through energy transfer between ^3^PS^*^ and ^3^O_2_ (Chen et al., 2021). It is also worth mentioning that both type I and type II photochemical reactions can occur simultaneously in a competitive manner (Li et al., 2022). The overall efficacy of PDT in both type I and II reactions, is dependent on three primary components: light, PS and O_2_. Unlike type I PDT photochemical reactions, emerging studies report type II photochemical reactions of having poor therapeutic efficacy in hypoxic cancer cells (Chen et al., 2021; Ma et al., 2022). Nevertheless, the mechanism of action of many PSs used in cancer treatment are based on induction of type II reactions (Chen et al., 2021; Li et al., 2022).

As discussed, among all the photogenerated ROS, ^1^O_2_ generated through type II energy transfer reaction is considered to be the major determinant of PDT induced cell killing (Sai et al., 2021). However due to high reactivity, short lifetime and diffusion distance in cellular conditions, ^1^O_2_ mediated PDT treatments are only effective within limited range (Kuimova et al., 2009). Thus, too little ^1^O_2_ fails to effectually treat solid tumor mass, while very high amount of generated ^1^O_2_ can damage and kill surrounding healthy cells leading to adverse effects. Presently, in clinical settings adjustment of delivered light intensity is done to control the extent of ^1^O_2_ generation. Nonetheless, this approach suffers from limitations whereby (Pavlova et al., 2022) higher light fluency rates lead to O_2_ depletion and PS photobleaching; and (Hanahan, 2022) long exposure time for low light fluency usually results into hypoxic condition due to vascular shutdown (Zhang et al., 2008). In this regard, over the past decades tremendous advancements in nanotechnology have led to the development of several nanomaterials-based PS (nano-PS) designing strategy in an effort to enhanced photostability, ^1^O_2_ generation efficacy. Among the several available nano-PSs, plasmonic metal nanoparticles (NPs) mediated enhancement in ^1^O_2_ generation is a promising approach which holds potential for significant improvement of future PDT (Yaraki et al., 2022).

Various novel approaches are actively being explored to overcome several of these issues. Over the past decades the era of nanotechnology has also opened new opportunities for extensive application of nanomaterial-based PDT agents for therapy as well as diagnostics. Although several reviews have extensively discussed the important concepts, strategies, significant advances and rationale behind the designing and applications of metallic NPs in PDT (Sai et al., 2021; Li et al., 2022; Ma et al., 2022). This article majorly focuses on potential role of plasmonic metallic NPs with or without PS in the enhancement of ^1^O_2_ generation as an approach for improved PDT of cancer. This article further discusses and provide an understanding of state-of-the-art designing strategy of plasmonic metallic NPs and underlying mechanism for boosted ^1^O_2_ generation. Additionally, this review briefly introduces the basic principles of PDT in cancer and importance of nano-PSs in PDT.

## 2 Nano-Photosensitizers

Nanotechnology is defined as the technology on the nanoscale, representing a modern interdisciplinary science including physics, chemistry, biology, and engineering, which have revolutionized the bio-nanotechnology and nanomedicine fields with its promising applications in health and medicine extending from prevention, and diagnosis, to the treatment of severe diseases such as cancer (Chang et al., 2015; Kargozar and Mozafari, 2018; Prasad et al., 2018; Yao et al., 2020). The unique chemical, electrical, structural, mechanical, magnetic, and biological properties of nanoscale-sized materials have found wide application in nanomedicine, from drug delivery, *in vivo* imaging and therapy microfluidics, biosensors, microarray tests to tissue engineering. Nano-based drug delivery systems are of major interest in bio-medics which make use of nanostructures as delivery vehicles by encapsulating or attaching therapeutic drugs and delivering them to target tissues with specificity and selectivity alongside various routes of administration. Furthermore, nano delivery systems reduce the toxic side effects and high administrative doses of drugs by enhancing the therapeutic activity by prolonging the half-life of drugs, improving the solubility of hydrophobic drugs, and reducing potential immunogenicity with more precise controlled release of drugs in a sustained or stimuli-triggered fashion (Lombardo et al., 2019). Since the development of first-generation PSs, the Food and Drug Administration (FDA) approved lipid-based nano-drug vehicles i.e., liposomes and micelles in the 1960s. Over a dozen of nanotechnology-based therapeutic products have been approved by FDA for both clinical trials and clinical applications (Shi et al., 2010; Patra et al., 2018; Sim and Wong, 2021).

**FIGURE 3:**
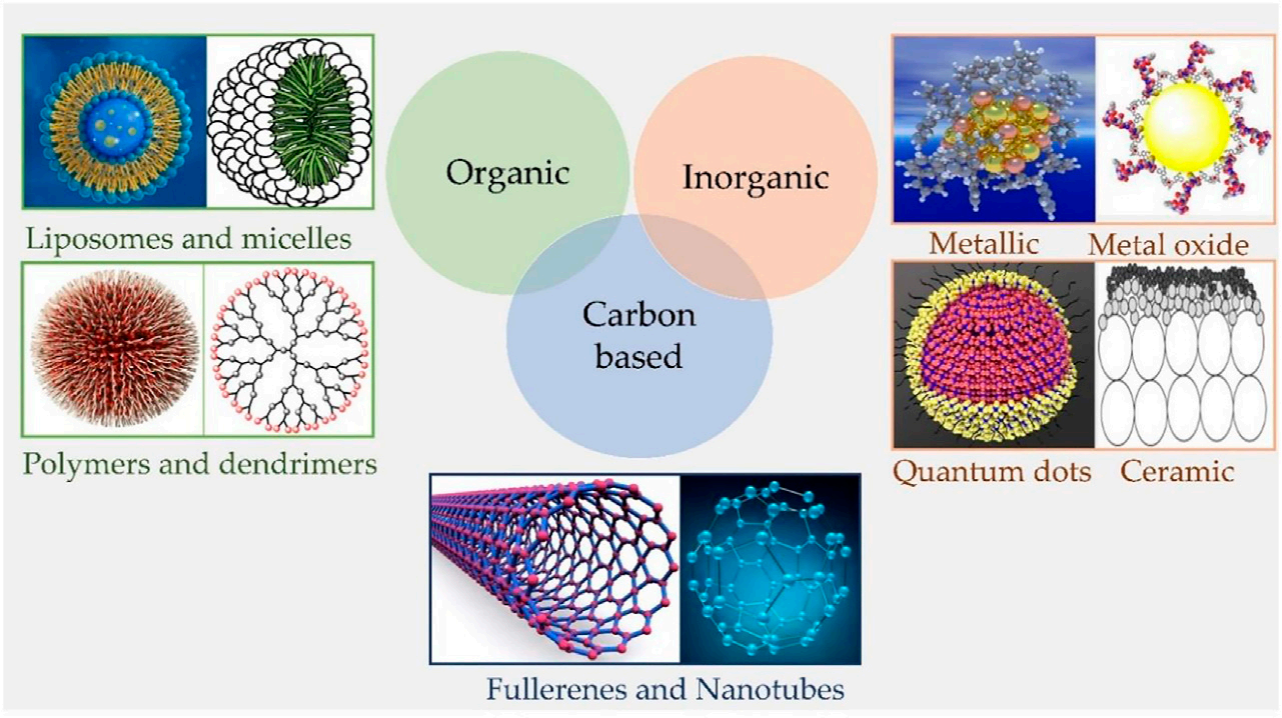
Schematic representation of different types of nanomaterials. Organic nanoparticles including Liposomes, micelles, dendrimers, and polymeric nanocarriers; Inorganic nanoparticles involve metallic, metal oxide, ceramic nanoparticles, and quantum dots; and Carbon-based nanoparticles such as Fullerenes and carbon nanotubes, etc.

The application of nanotechnology in PDT started with the first report by Labib et al., in 1991, whereby they showed the synthesis of cyanoacrylic nanocapsules (150–250 nm) and NPs (10–380 nm) encapsulated with phthalocyanine or naphthalocyanine derivatives (Labi b et al., 1991). Importantly, the encapsulated or loaded PS in the nanocarrier does not need to be released from the carrier as both molecular O_2_ and generated ^1^O_2_, are able to diffuse in and out of the nanocarrier. In contrast to chemotherapeutic drugs. This widened the use of several different materials and strategies for developing nano-PS (Figure 3), and generally are sub-classified as: 1) Biodegradable NPs which includes natural or synthetic polymer-based NPs, which usually undergo *in vivo* enzymatic or hydrolytic degradation and thus are easily excreted out to minimize their long-term accumulation and toxicity or 2) nonbiodegradable NPs based on silica, ceramic and metals, which does not readily degrade in the biological system, but offers the advantage of easy control of particle size, shape, porosity, and monodispersibility and have multiple functionalities, making them valuable as theranostic agents (Huang et al., 2013; Lucky et al., 2015). The significance and broader applications of nanotechnology have also widened its scope into PDT for utilization of nano-PS for therapy and diagnostics, as well as overcoming its several limitations (Olivo et al., 2010; Lucky et al., 2015; Xie et al., 2021; Lee et al., 2022). Compared to the free molecular conventional PSs, nano-formulations of PSs impart certain unique and improved properties that make them more potent for PDT applications. First of all, nanomaterials act as a stable and potent drug delivery carrier which generally overcomes the shortcomings of free molecular PSs like hydrophobicity, aggregations, and low tumor cell/tissue specificity, and further permits higher loading of PSs allowing controlled delivery and accumulation in tumor mass in higher concentrations (Zhang et al., 2016; dos Santos et al., 2021; Yan et al., 2020). The smaller size and stability of NPs prolongs the circulation of PSs in the system, enables passive targeting and higher accumulation of PSs in the tumor mass by enhanced permeation and retention (EPR) effect, whereby the leaky and improperly generated tumor vasculatures allow enhanced penetration of circulating NPs in tumor tissues. Moreover, tumor accumulation of PS-loaded NPs can be further improved by active targeting through surface modifications of NPs with specific cancer-targeting ligands (dos Santos et al., 2021; Yan et al., 2020; Zarrintaj et al., 2018).

Additionally, selective accumulation of nano-PSs in higher concentration is also highly desirable to concentrate the PDT-induced ROS generation in tumor cells/tissues, due to the short lifetimes and diffusion distance of ROS under physiological conditions. The major PDT-generated ROS i.e., ^1^O_2_ has a lifetime of 15–30 μs with the propagation distance up to 0.5–1 μm only (Peskova et al., 2021). Similarly, all other free radicals like O_2_^•−^ and OH^•^ ions being highly reactive have a short lifetime and mean distance in cells. While contrarily, hydrogen peroxide being uncharged and more stable has a longer life up to 1 ms in cells and can traverse membranes freely, and is majorly involved in the PDT-mediated oxidation of membrane lipids and proteins in both primary and secondary response to irradiation of PSs (Peskova et al., 2021; Anjum et al., 2022). This eventually aggravates PDT-induced cell death and subsequent response (Peskova et al., 2021). Secondly, promising optical properties of several nanomaterials, especially metallic and semiconductor NPs, have been explored to modulate several photophysical properties of nearby PSs such as enhancement of light absorption efficiency, cross-section area, NIR excitation, fluorescence property, and energy transfer or electron transfer process to augment ROS generation to achieve improved penetration and efficacy of PDT (Wang et al., 2020). These properties are mainly offered by plasmonic metal NPs, aggregation-induced emissive nanodots, semiconductor NPs carbon-based quantum dots, Lanthanide doped upconversion NPs and ultrasmall metal nanoclusters (<2 nm) which are widely being explored for ROS enhancement, and advanced PDT approaches such as upconversion PDT, Two Photon PDT and Self-illuminated PDT (Zhang et al., 2016; dos Santos et al., 2021). However, as compare to noble metal NPs, semiconductor NPs like Quantum Dots and upconversion NPs with heavy elements and lanthanides, raises the health hazard concerns regarding their pharmacokinetic pharmacodynamic properties which such as their ultimate fate in the biological system, possibility of their degradation into toxic byproducts, and their short and long term effects (Lucky et al., 2015).

## 3 Metal nanoparticles

Metal-based nanomaterials are mainly categorized into 1) metal NPs i.e., pure forms of metal-based NPs, e.g., silver, copper, gold, titanium, platinum, zinc, magnesium, iron NPs, 2) metal oxide NPs such as titanium dioxide, silver oxide, zinc oxide, etc., 3) doped metal/metal oxide/metal nanomaterials and 4) metal sulfide and metal organic frameworks (MOFs) nanomaterials, e.g., AgS, CuS, FeS NPs, Zn-based MOF, Cu-based, Mn-based MOF, etc. (Yaqoob et al., 2020). Extensive discussion on important concepts, strategies, significant advances, and rationale behind the designing and general applications of metallic NPs in PDT is beyond the scope of this review and has already been extensively reviewed (García Calavia et al., 2018; Sun et al., 2018; Khursheed et al., 2022).

Importantly compared to free form of PSs, other than acting as drug carrier and inducing EPR effect, plasmonic metal NPs with or without PSs impart the advantages of 1) energy transducer whereby their large extinction coefficients about 5 orders of magnitude larger allows efficient energy transfer process for photosensitization thus needs lower laser energy to trigger PDT without damaging the nearby healthy cell, 2) enhance ^1^O_2_ generation of PS based on a physical phenomenon called metal-enhanced ^1^O_2_ generation 3) tuning the Surface Plasmon Resonance (SPR) in NIR wavelength region for deep tissue penetration (Ho-Wu et al., 2017; Younis et al., 2021; Yaraki et al., 2022). Metallic NPs may be synthesized and manipulated such that they can attach to antibodies, drugs, and ligands (Sharma et al., 2015; Venkatesh, 2018). Thus, with all these properties plasmonic metal NPs are best used for enhancing conventional PSs. In the following section, we have highlighted and signified the importance of the fundamental knowledge and mechanistic pathways of photo-nano-chemistry of plasmonic metallic NPs for a better understanding of the enhancement strategies for ^1^O_2_ generation.

### 3.1 Plasmon resonance induced properties in noble metal nanoparticles

**FIGURE 4:**
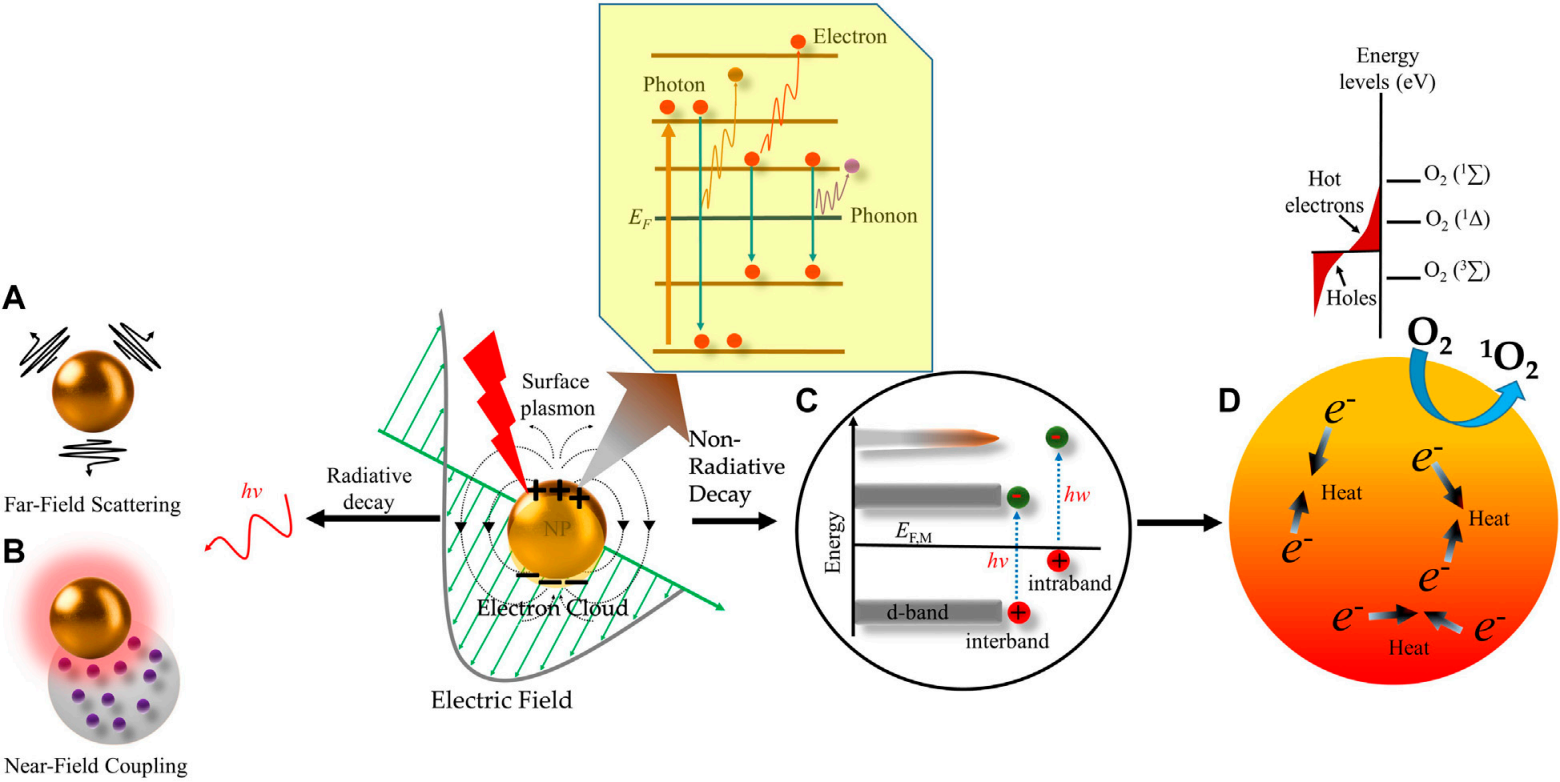
Schematic representation of radiative and non-radiative decay of plasmons in metal nanoparticles. Excitation of plasmonic nanoparticles (NP) generates localized surface plasmon resonance (LSPR). Surface plasmon decays in three different ways, i.e., electron-to-photon, electron-toelectron, and electron-to-phonon (subset). The radiative decay process either gives rise to **(A)** light scattering into the nanostructures’ “far-field.” i.e., a region that is far from the electromagnetic field, or **(B)** electromagnetic fields in the nanostructures’ “near field”, i.e., a region that possesses inductive effect. The nonradiative decay proceeds through **(C)** intraband excitation within the conduction band of the metal or an interband excitation between occupied bands (usually d bands) and the conduction band. This results in the generation of **(D)** non-thermal and “Hot electrons” which have high energies to excite molecular oxygen (O_2_) to its excited state to generate singlet oxygen (^1^O_2_). The scattering of Hot electrons and phonons within the NP increases its temperature by transferring their excitation energy to the NP lattice, which is further transferred to the surroundings by heat conduction and ultimately generates local heating effect.

Among all the nanomaterials, metallic NPs have unique optical properties due to their ability to interact with an incident of electromagnetic radiation. These optical properties include extinction, absorption, Rayleigh scattering, and Raman scattering, and electronic property like conductivity along with the biocompatibility of metallic NPs make them a promising candidate for therapeutic as well as diagnostic applications (Krajczewski et al., 2019; Yaqoob et al., 2020; Khursheed et al., 2022). However, the optical properties of metals depend greatly on their size. Where the nanoscale metals (<5 nm) have a continuous band of energy levels i.e., an overlap between the valence and conduction bands: the outer valence electrons move around freely as conduction electrons within the metal, which respond efficiently to outside perturbations, such as electromagnetic fields. Under illumination, the oscillating electric field of the electromagnetic wave completely permeates the NP which perturbs the conduction electrons within the whole volume of the NP’s (Carrasco et al., 2020). Initially, the electrons coherently couple to this oscillating electric field and form an electron density or cloud. Once the wavelength of the incident light larger than the size of metallic NP is illuminated, the electron cloud decreases on the illuminated side of the NP and increases on the other opposite side resulting in an asymmetrically distributed electron cloud. This leads to a series of oscillations in the vicinity of the metallic NP creating an opposing electric field to the externally applied electric field. The resulting coherent/collective oscillations of the charge density and the corresponding electric field are denoted as localized surface plasmons (LSPs) as shown in Figure 4. These electronic oscillations produce two modes: 1) surface plasmon–polariton (SPP), which propagates along with the metal/dielectric interfaces, and 2) localized surface plasmon resonance (LSPR), which is confined in a very small volume around the isolated metallic NP. In general, the specific frequency at which the amplitude of the oscillation reaches the maximum is referred to as plasmon resonance frequency that leads to a LSPR. During LSPR, the electric field of incident light periodically displaces the NP sphere’s electrons with respect to the lattice ions, generating the oscillating electron density. Excitation of LSPR causes nanoscale localization and enhancement of electromagnetic fields in the vicinity of the metal NPs. As shown in Figure 4, the LSPR electrons strongly absorb and scatter light at the LSPR wavelengths of plasmonic-metal NPs in both visible and near-infrared ranges (Kim et al., 2017). NPs of smaller than 15 nm, spectral resonance is dominated by absorption, while larger NPs of >15 nm have a scattering-dominated spectral resonance (Li et al., 2015).

Further, due to the electron-electron and electron-phonon interactions, the resulting decoupling of the electronic cloud from the oscillating electric field promotes a particular subpopulation of conduction electrons to a high-energy state known as non-thermal electrons (Figure 4C). Non-thermal electrons have energy significantly above the Fermi level, a threshold energy level for electrons to engage in chemical reactions or leave the particle (ionization). Due to the electron-electron scattering effect, the non-thermal electron population becomes unstable for further thermalization, and the excess energy is redistributed among all the electrons in the particle, leading to hot electrons (Figure 4C). The hot-electron population having a longer lifetime is responsible for chemical reactivity after plasmon excitation. Finally, as shown in Figure 4D, due to the electron-electron and, electron-phonon scattering the heat from the hot-electron is redistributed to the whole NP and increases its overall temperature resulting in photothermal effect (Kim et al., 2017; Carrasco et al., 2020). Most importantly, the rationale for designing plasmonic metallic NPs with desirable plasmonic properties i.e., LSPR and generated thermal energy is directly correlated with fine-tuning of their optical response which is greatly influenced by several factors such as the size and shape of metal NPs, the configuration of metal NPs, the wavelength of the incident light, dielectric function of the NPs and dielectric constant of the surrounding medium (Kelly et al., 2003; Khlebtsov and Dykman, 2010; Rycenga et al., 2011; Manzhos et al., 2021). Further, usually AgNPs and AuNPs show the LSPR band at 390 nm and 520 nm in the absorption spectra, respectively (Jain et al., 2008; Tong et al., 2009; Huang and El-Sayed, 2010). As a general rule, as the size of NPs increases, the LSPR peak in absorption spectra becomes more red-shifted. The Ag nanospheres solutions with a different mean diameter of 3.1 ± 0.6, 13.4 ± 5.8, 46.4 ± 6.1, and 91.1 ± 7.6 nm showed their LSPR peak wavelength at 390 nm, 393 nm, 408 nm, and 440 nm with a shoulder peak at 384 nm, respectively (Nallathamby et al., 2010). Similarly, an increase in the size of AuNPs from 19 to 66 nm–106 nm shows a gradual red shift in LSPR wavelength, from 520 to 527 nm–531 nm respectively (Khaing Oo et al., 2012). Further, Li et al., demonstrated that the LSPR absorption peak varies for three AuNP shapes. The 15 nm diameter nanospheres showed a characteristic LSPR absorption at 520 nm, the nanorods of 30.2 nm length and 9.3 nm diameter exhibited transverse and longitudinal LSPR peaks at 515 and 735 nm, respectively and the nanostars with a core of 34.5 nm diameter displayed two LSPR bands at 532 and 675 nm (Li et al., 2012). Very few metals can act as potent plasmonic NPs in the visible region for example Li, Al, Cu, Pd, Ag, Pt, and Au. However, each metal has its own advantages and disadvantages for plasmonic applications based on its plasmonic resonance, chemical stability, nanostructure formation, and cost. For example, Ag has the strongest resonance across most of the spectrum from 300 to 1200 nm followed by Au and Cu which have LSPR excitation wavelengths longer than 500 and 600 nm, respectively. However, Cu nanostructures impose the concern of instability and toxicity for biological applications. Pt and Pd have the weakest resonance property and being the costliest among all, makes them unsuitable for large-scale applications. Al is only preferable for UV region applications and the high reactiveness of Li makes it very difficult to handle as NPs. Considering all these factors, noble metals Au and Ag are the most promising as plasmonic NPs for *in vivo* applications. However, surface modification of Ag nanostructures is needed to enhance their biocompatibility and stability and attenuate their toxicity by eliminating the release of Ag^+^ ions. The bio-inertness, negligible reactivity, and ease of synthesis of Au nanostructures make them the most well-suited (Rycenga et al., 2011). Plasmonic NPs have extended biomedical applications, starting from biosensing, bioimaging, and drug delivery to photothermal therapy and PDT.

### 3.2 Role of plasmonic metal nanoparticles in PDT

In recent years metal NPs have gained tremendous interest in health sciences because of their conspicuous properties that are useful for the diagnosis and treatment of several diseases (Jamkhande et al., 2019). Common organic PSs e.g., silicon phthalocyanines and porphyrins, have been reported of having limitations such as lower molar extinction coefficients, poor photostability, poor enzymatic degradation, and inability to be activated by NIR light. This is because UV-Vis spectrum has poor tissue penetration depths, which overall restricts the use of NIR light-activated organic PSs in PDT with few exceptions (Vankayala et al., 2014a; Vankayala and Hwang, 2018; Lv, Zhang, Li, Wang, He; Sarbadhikary et al., 2021). The breakthrough observation of plasmon-mediated electron emission from the metallic NPs mainly gold and silver, into the surrounding media leading to ^1^O_2_ generation upon direct light irradiation made them a potential PS candidate for PDT (Lv, Zhang, Li, Wang, He). Furthermore, as discussed, metallic NPs offer several advantages over organic PSs of high stability, high loading or conjugation efficiency, adjustable size, optical properties, easy surface functionalization, slow degradation, and long cycle time, making them more biocompatible and resistant to decomposition in biological applications, which allows tumor targeting, delivery and controllable release of PSs (Sun et al., 2018; Shang et al., 2021). The metallic NPs exhibit high extinction coefficient which generally compensates for the somewhat low ^1^O_2_ yield and reduces the overall excitation power, thus, slowing down the depletion of tissue oxygen and promoting the reperfusion of tissue oxygen. Additionally, the feasibility of synthesis and functionalization of metallic NPs can easily tune their excitation wavelengths to the near-infrared (NIR) region, which in turn significantly enhances the penetration depth into the tissues and improve the overall *in vivo* PDT potential (Lv, Zhang, Li, Wang, He). Besides, these metallic NPs improve the overall efficacy of PDT via different phenomena like (Pavlova et al., 2022) efficient energy transfer from excited plasmonic metallic NPs either to molecular O_2_ and/or to certain standard organic PSs due to a FRET mechanism, and (Hanahan, 2022) light upconversion, where excitation of upconversion NPs by near-infrared radiation and emission of shorter wavelength light leads to the excitation of the organic PSs (Bucharskaya et al., 2016; Krajczewski et al., 2019; Sarbadhikary et al., 2021).

#### 3.2.1 Free noble metallic plasmonic nanoparticles

Among all the metallic NPs, AuNPs have been most extensively used in PDT, because of their special property of LSPR which involves heating of AuNPs by the application of light of a specific wavelength to the surface of the AuNPs. As discussed, LSPR is an optical phenomenon that occurs when light interacts with conductive metallic NPs that are smaller than the incident wavelength. AuNPs with various shapes and sizes such as nanocage, nanoflower, nanoshell, nanosphere, nanorod, nanostar, and nanoporous Au disks. The specific wavelengths, emission frequencies, and emission wavelengths of different AuNPs are highly dependent on the size, shape, surface, and aggregation state of the NP’s (Petryayeva and Krull, 2011; Kim and Lee, 2018). The LSPR property of AuNPs allows rapid energy transfer from Au metal surface to molecular O_2_ with high efficiency and forms ^1^O_2_, thus inducing PDT even without the involvement of PS (Krajczewski et al., 2019). As discussed above both the initial nonthermal electrons, and later hot-electrons have enough energy to directly pump jump to the higher electronic levels of O_2_ molecules generating ^1^O_2_ by energy transfer with these hot-electrons (Carrasco et al., 2020). Gao et al., discussed the mechanistic insights of plasmonic metal NP mediated generation of ROS under NIR one/two-photon irradiation for PDT occurs via energy and electron transfer modes. Photoexcitation of surface plasmons on AuNPs first decay into hot electrons with energies between the vacuum level and Fermi level of the metal. The hot electrons with high-energy levels further transfer into and populate the 2π* antibonding O–O orbital, creating a fleeting negative ion, O_2_^•−^, which subsequently relaxes by releasing an electron back to the metallic NP surface to create ^1^O_2_. Further, it was shown that Au nanocages showed almost 6-fold increase in ^1^O_2_ generation capability under two-photon irradiation compared to a one-photon irradiation (Gao et al., 2014).

In 2006, George Pasparakis demonstrated the principle of producing ^1^O_2_ and ROS by irradiating AuNPs articles using continuous-wave and pulsed laser sources. Where it was shown that two different underlying photochemically and/or photothermally reactions are involved in AuNPs mediated cancer cell killing upon laser irradiation. First, one plasmon-activated pathway involves interactions of plasmons and hot electrons with molecular oxygen, and secondly an indirect photothermal pathway that induces the generation of extreme heat leading to particle fragmentation and increased thermionic electron emission, where both the pathways. The cancer death in the case of irradiation of AuNPs with continuous-wave and pulsed laser sources exhibited that generation of ^1^O_2_ and ROS significantly amplified the overall *in vitro* cancer cell death during photothermal and photodynamic treatment (Pasparakis, 2013).

For the first time, Vankayala et al. demonstrated that naked AuNPs alone have the potential to generate ^1^O_2_ to exert PDT effects. Au nanorods were shown to completely destroy melanoma tumors in mice upon irradiation with NIR 915 nm light via PDT effect which was far more effective than the AuNP mediated photothermal therapy (PTT) effect at 780 nm light excitation. Moreover, these NPs induced ∼10-fold higher PDT cell death in HeLa cells irradiated with 940 nm (Vankayala et al., 2014a). Multi‐branched Au nanoechinus structure with exceptionally high extinction coefficients in the NIR region (800∼1700 nm) exhibited dual-modal *in vivo* PDT and PTT effects upon excitation with 915 nm (NIR I) and 1064 nm (NIR II) for the complete destruction of solid tumors in melanoma mice model. NIR activation of Au nanoechinus treated cancer cells at 940 nm demonstrated ∼2.5 folds ^1^O_2_ generation when compared to 550 nm irradiation as well as dark (Vijayaraghavan et al., 2014). Several studies involving naked unmodified Au spheres, Au nanorods, Au bipyramids, Au nanocages, spherical hollow AuNPs, and Au nanorods in shells have also been shown to be potential materials for inducing ^1^O_2_ mediated PDT. These nanomaterials exhibit the maximum ^1^O_2_ generation efficiency when the incident laser wavelength overlaps with their SPR peaks (Lv, Zhang, Li, Wang, He; Vankayala et al., 2014b).

Further, it was shown that the ^1^O_2_ formation by metallic NPs is strongly dependent on their morphology. This was evident from the observation that ^1^O_2_ was generated by photo-irradiating silver nanomaterials of decahedrons and triangular plate morphology, but not by Ag nanocubes. *In vitro* studies exhibited ∼4-fold higher photo-induced cellular deaths in HeLa cells with Ag decahedral NPs than Ag nanocubes upon irradiation with a 940 nm NIR light (Vankayala et al., 2013).

Other than the free AuNPs, their aggregates have been also evaluated as PSs for PDT. Where it has been shown that aggregation of AuNPs usually significantly increases the efficiency and yield of ^1^O_2_ generation than for the isolated unaggregated forms (Han et al., 2012; Jiang et al., 2013). For example, two-photon induced ^1^O_2_ generation capabilities of aggregated Au nanospheres and short Au nanorods were 15.0- and 2.0-fold higher respectively compared to their unaggregated state (Jiang et al., 2013). Direct photosensitization of Au clusters like organic-soluble Au25 (Phenylethanethiolate) 18– and water-soluble Au25 (Captopril)18– have also been demonstrated to efficiently produce ^1^O_2_ under visible/near-IR (532, 650, and 808 nm) irradiation. Photoexcitation of water-soluble Au25 (Captopril) 18– clusters at 808 nm induced photodynamic cell killing of cancer cells (Kawasaki et al., 2014). Usually, metal nanoclusters of less than 2 nm in size do not have LSPR, but rather, they exhibit discrete optical transitions (Díez and Ras, 2011; Jin and Higaki, 2021). They have several valuable properties for PDT application which include long-lived triplet excited states, absorption cross-sections, high photostability, small metal clusters that are ideal for deeper penetration in the cells, and ^1^O_2_ generation efficiency. Ho-Wu showed the ^1^O_2_ generation capacity of three different metal nanoclusters in the order of Au144 > Au25, Ag32, which was several orders of magnitude higher than plasmonic Au NPs (40 nm). This increase in ^1^O_2_ production is attributed to the high absorption cross section-to-volume ratio in nanoclusters resulting in enhanced triplet excited states population (Ho-Wu et al., 2017).

Other, than free plasmonic metal NPs, the approach of incorporation of plasmonic metallic NPs into inorganic semiconductors such as TiO_2_ have been explored for plasmon-enhanced ^1^O_2_ generation under visible light irradiation. This overcomes the limitations of inorganic semiconductor NPs which can only be excited by UV light illumination, which restricts their application for *in vivo* PDT. The proposed mechanism involves LSPR-induced generation of high-energy (hot) electrons in metallic NPs upon photoexcitation of metal-semiconductor nanostructures with visible light, these hot electrons *via* the electron transfer process are then transferred to the conduction band of semiconductor NPs. Subsequently, the electrons in the semiconductor conduction band reduce the surrounding O_2_ molecules to O_2_^•−^, which later oxidizes to ^1^O_2_ by the holes remaining in metal NPs or returns to the ground state O_2_ (Zhou et al., 2015). Some reported examples of such metal-semiconductor hybrid nanostructures include AuNPs deposited ZnO NPs (He et al., 2014), AuNP-deposited TiO_2_ (Saito and Nosaka, 2012), AuNP core/TiO_2_ shell nanostructures (Fang et al., 2014) have shown plasmon-enhanced photocatalytic ^1^O_2_ generation.

#### 3.2.2 Hybrid photosensitizer-plasmonic metallic nanoparticles

Other than acting as efficient PS carriers, plasmonic metallic NPs in proximity to traditional PSs have shown to exhibit strong light absorption in the NIR region and high ^1^O_2_ generation capability. The effect is due to the plasmon-molecular resonance coupling, which occurs when a light-absorbing molecule is placed near plasmonic metal NPs. The coupling leads to the formation of hybrid states having spectral properties different from those of the individual constituents this occurs due to the hybridization of the plasmon and molecular resonance (Zhang et al., 2008; Hu et al., 2014).

Wang et al. demonstrated the efficacy of hybrid nanostructures consisting of plasmonic Ag NPs coated by Hematoporphyrin IX-loaded mesoporous silica (Ag@mSiO_2_@HPIX) with significant enhancement in ^1^O_2_ production. These hybrid PS-metal NPs induced efficient phototoxicity against cancer cells upon exposure to red/near-infrared light excitation, thus effective for deep-tissue cancer treatments. Excitation of Ag@mSiO_2_@HPIX hybrids with 400 nm exhibited a higher ^1^O_2_ generation with an enhancement factor of 3.1 compared to free HPIX or Ag@mSiO2 separately. Moreover, as HPIX have no ^1^O_2_ production under NIR excitation while irradiation of Ag@mSiO_2_@HPIX hybrids at 850 nm resulted in significantly higher production of ^1^O_2_ of 4.2 enhancement factor (Wang et al., 2016). The ^1^O_2_ quantum yield was measured to be higher for Zinc-hexadecafluoro-phthalocyanine coated AuNPs (0.65) compared to the value of 0.45 for free Zinc-hexadecafluoro-phthalocyanine (Hone et al., 2002).

Similarly, hybrid PS- metallic nanocarriers like AuNP-core with SiO_2_-shell incorporated with methylene blue (MB) (Chu et al., 2013), silica-coated Au nanostars embedded with MB (Fales et al., 2011), silica-coated AuNRs linked with tetra-substituted carboxyl aluminum phthalocyanine (AlC_4_Pc) (Ke et al., 2014), 5-aminolevulinic acid-conjugated AuNPs (Khaing Oo et al., 2008), phthalocyanine conjugated AuNP (Wieder et al., 2006), exhibited Au plasmonic effect for enhanced ^1^O_2_ generation efficacy resulting into enhanced PDT effect. The capability of plasmonic NPs to generate ^1^O_2_ is also shown to vary with the distance between PS and the metallic surface of NPs. For example, ^1^O_2_ generation of AlC_4_Pc was reported to be highest for AuNRs@SiO_2_-AlC_4_Pc with the silica shell thickness of 10.6 nm between AlC_4_Pc and Au nanorod surface, when excited with 680 nm light (Ke et al., 2014). de Melo et al. showed that excitation with 415 nm light, riboflavin-silver nanospheres of 13 nm, coated without and with a pectin layer resulted in a 1.4 and 1.8-fold enhancement in photogenerated ^1^O_2_ generation respectively, compared to the free riboflavin solution (De Melo et al., 2012). In another study, Protoporphyrin IX (PpIX) coated Ag core SiO_2_ shell NPs demonstrated that the ∼100 nm Ag core surrounded by the thinnest SiO_2_ shell of ∼5 nm induced the highest enhancement in photogenerated ^1^O_2_ up to 5 times compared to PpIX-functionalized SiO_2_ NPs. This is attributed to the efficient excitation of the LSPR of Ag NPs and overlap between PpIX absorption band and the excitation of Ag NP LSPR (Lismont et al., 2016a).

Another study reported a nanosystem composed of mesoporous silica-coated Au nanorods incorporated with indocyanine green (ICG). The LSPR peak of the Au nanorod core was fine-tuned to overlap with the absorption band of ICG, which by maximizing the local field enhancement of AuNR, improves the absorption coefficient of incorporated ICG and protecting the ICG molecules against photodegradation thus eventually showing strong augmentation in ^1^O_2_ yield without changing ICG payload. Further, the incorporation of ICG in the silica shell led to the formation of ICG aggregates having high photostability and thermal stability than ICG monomers. The reported designing principle enhanced the antitumor efficacy of ICG against human breast carcinoma cells in both *in vitro* and *in vivo* models, through a synergistic effect of plasmonic-PDT and photothermal therapy. Further, ^1^O_2_ production for the Au@SiO_2_-ICG after 1-, 2-, and 3-min irradiation with 808 nm laser was observed to be 1.5, 3.6, and 6.3 times respectively with respect to free ICG. Additionally, the nano platform facilitated trimodal imaging with ICG-mediated NIR fluorescence and two-photon luminescence/photoacoustic tomography due to AuNRs (Li et al., 2014). Extending the applications of plasmonic metallic NPs, a nanocomposite consisting of a core of NaYF4:Yb/Er upconversion NPs conjugated with Au nanorods and coated with a silica shell embedded with methylene blue (MB) was evaluated for its plasmon-enhanced PDT efficacy. Hereby, the efficacy of ROS generation by 980 nm irradiation of UCP@SiO_2_:MB was observed to vary with the thicknesses of the SiO_2_ shell as 4.5 > 8.2>1.5 > 13.2 nm. Thus, UCPs served as a light converter NIR to visible light to excite MB, and AuNRs effectively enhanced the upconversion efficiency and ROS generation *via* the LSPR effect and exhibited efficient photodynamic ability both *in vitro* and *in vivo* oral cancer model (Chen et al., 2016). As a proof of principle Oo et al. assessed the correlation of enhanced ^1^O_2_ generation with the particle size of metallic NPs which in turn controls the LSPR field. Whereby, PpIX was conjugated with AuNPs of 19, 66, and 106 nm diameter and investigated for their PDT efficacy against breast cancer cells *in vitro* study. The results revealed that the 66 nm PpIX-AuNPs induced the highest PDT-mediated cell destruction, consistent with their highest cellular uptake and ^1^O_2_ production. Herein, different sizes of Au NPs also influenced the ROS enhancement ratio by 1:3.33:11.65 for 19, 66, and 106 nm, respectively (Khaing Oo et al., 2012). A nanocomposite design composed of silica-coated Au–Ag nanocage core functionalized with Yb–2,4-dimethoxyhematoporphyrin (Au-Ag/SiO_2_/Yb-HP) also demonstrated efficient generate on of ^1^O_2_ and enhanced cancer cell killing simultaneously with IR-luminescence imaging (Khlebtsov et al., 2011). Table 1 shows common metal NP-PSs for enhanced PDT with variations in photochemical properties.

**TABLE 1:** A summary of examples of metal nanoparticles (NPs) in combination with photosensitizers (PS) and their photophysical properties and PDT enhancement efficacy.

Complete NP	Metal	PS	λmax (nm)	^1^O_2_ quantum Yield (a.u)	Enhancement Factor/ratio	Cancer Cell line	IC_50_ (μM)	Ref
Ag@mSiO_2_@HPIX	Ag	HPIX	850	∼3.2	4.2	Hela	-	Wang et al. (2016)
ZnPcF_16_ AuNPs	Au	ZnPcF_16_	695	0.65	1.4	-	-	Hone et al. (2002)
AuNRs@SiO_2_-AlC_4_Pc	Au	AlC_4_Pc	680	∼3	∼2.1	-	-	Ke et al. (2014)
Rf-Pec@AgNP	Ag	Rf	415	1.8	0.7	Hela	50	(De Melo et al., 2012) (Rivas Aiello et al., 2018)
AgSiO_2_-PpIX NP	Ag	PpIX	480–630	∼5.5	∼5.5	-	-	Lismont et al. (2016b)
Au@SiO_2_-ICG	Au	ICG	808	∼1.5 × 10^3^	6.3	MDA-MB-231	-	Li et al. (2014)
UCP@SiO_2_:MB	UCP	MB	980	∼1	∼10	OECM-1	-	Chen et al. (2016)
PpIX-AuNP	Au	PpIX	485	∼2.69 × 10^3^	11.65	MDA-MB-231	-	Khaing Oo et al. (2012)

## 4 Conclusion

**FIGURE 5:**
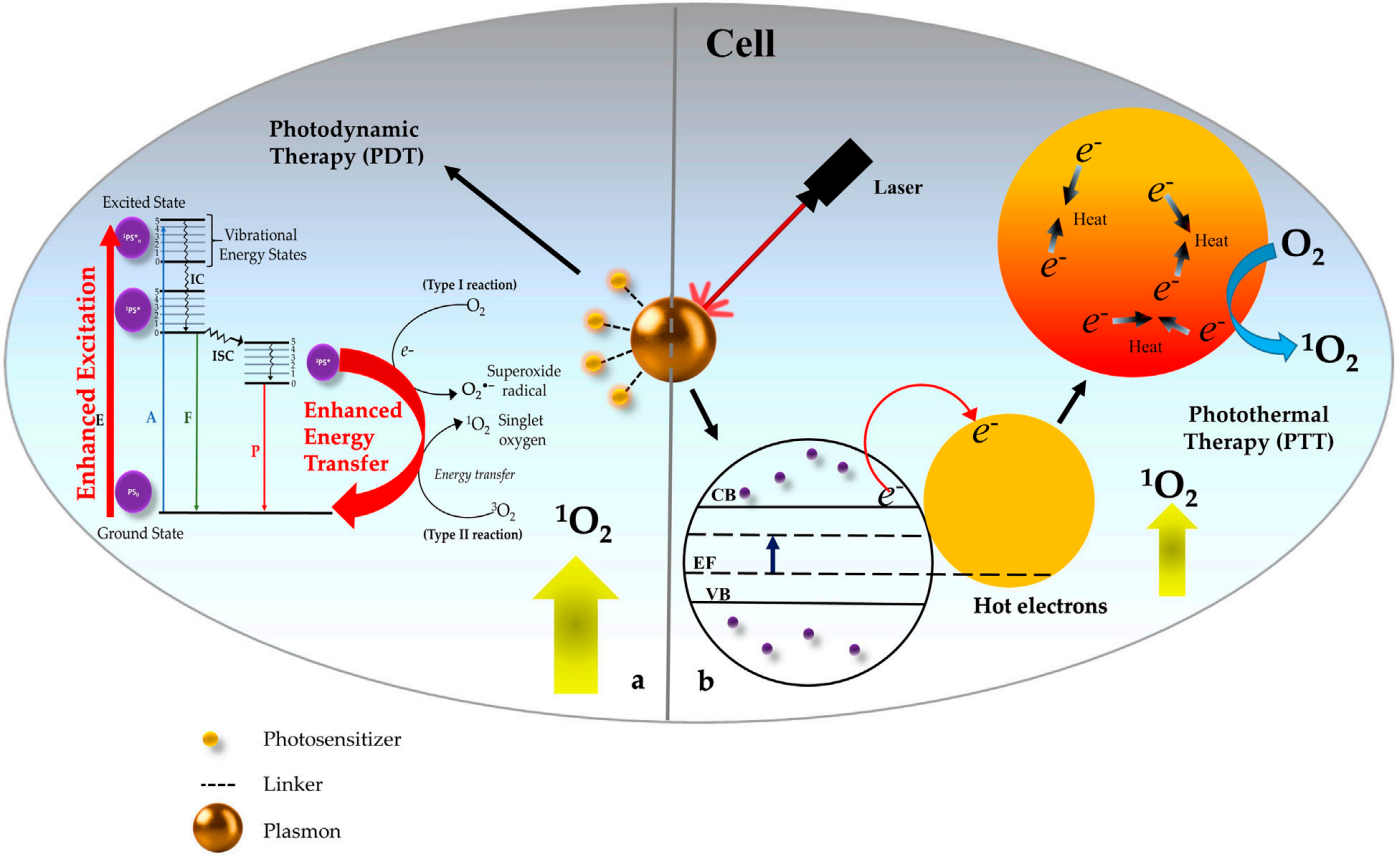
Schematic representation of Photothermal Therapy (PTT) and Photodynamic Therapy (PDT) effects of metal plasmonic nanoparticles (NP) within the cell. **(A)** Excitation of NP in combination with Photosensitizer (PS) causes photosensitized singlet oxygen (^1^O_2_) production through surface plasmon-photosensitizer (PS) resonance coupling which occurs due to enhanced excitation in the conjugates with good spectral overlap between the surface plasmon of NPs and the absorption of the PS. **(B)** Excitation of NP without PS majorly results into PTT due to local heating effect and moderate level of (^1^O_2_) generation due to Hot electron transfer within the plasmonic NP structure.

In recent years PDT is gaining a prominent interest among clinicians and patients, because of several improvements in the advanced PS designing and delivery strategies, technology, and optoelectronic equipment, and overcoming the limitations of PDT. As discussed in this review nanotechnology is one the field which has proven to be a very promising approach for future advancements and applications of clinical PDT. Among several different NPs, plasmonic metallic NPs exhibit a number of desirable and unique properties for use in PDT including providing a good biocompatible PS carrier, enhancing the ^1^O_2_ generation in conjugation with PS, increasing the fluorescence property of PS for fluorescence imaging, and shifting the excitation wavelength from visible to NIR region along with enhancing the absorption coefficient of PS for deep cancer treatment. This approach offers the flexibility of using any type of PSs which relies on the interactions of light with the plasmonic metal NPs to act as an efficient energy transducer for the PS that are placed in close proximity. Thus, plasmonic engineering strategy is a promising approach to enhance the ^1^O_2_ generation efficiency of PSs, rather than developing new more potential PSs. Moreover, such metal-enhanced nano-PSs exhibit the property of higher photostability and minimum photobleaching, making them a potential theranostic agent for image-guided therapy. Figure 5 represents a schematic illustration of the best-known mechanistic insights of PTT and PDT effects of metal plasmonic NP within the cell ultimately leading to cancer cell death.

However, efficient plasmonic metal NP enhanced ^1^O_2_ generation phenomena is dependent on several factors like size, shape, composition, the distance of PS from metalcore, excitation wavelength, and even metal NPs-PS molar ratio. would affect the final SOG enhancement factor. Thus, future work should be directed toward determining the optimized conditions and effective parameters for different plasmonic NP-PS formulations. Besides the promising properties of metallic NPs, it is of utmost importance to carefully examine the long-term toxicity, pharmacokinetics, and pharmacodynamic properties of metallic nanocarriers to avoid their unnecessary liver and kidney accumulation. Therefore, it is expected that plasmonic NPs and plasmonic composites will have a tremendous impact on the detection and treatment of cancer in the near future provided their inherent toxicity issues are taken care of before proceeding into clinical trials.
